# Analysis of Cleavage Activity of Dengue Virus Protease by Co-transfections

**DOI:** 10.21769/BioProtoc.4946

**Published:** 2024-03-05

**Authors:** Lekha Gandhi, Musturi Venkataramana

**Affiliations:** Department of Biotechnology and Bioinformatics, School of Life Sciences, University of Hyderabad, Hyderabad, Telangana State, India;

**Keywords:** Dengue virus, Protease, Co-transfection, Flavivirus, Anti-protease molecules, Western blotting

## Abstract

The genome of the dengue virus codes for a single polypeptide that yields three structural and seven non-structural (NS) proteins upon post-translational modifications. Among them, NS protein-3 (NS3) possesses protease activity, involved in the processing of the self-polypeptide and in the cleavage of host proteins. Identification and analysis of such host proteins as substrates of this protease facilitate the development of specific drugs. In vitro cleavage analysis has been applied, which requires homogeneously purified components. However, the expression and purification of both S3 and erythroid differentiation regulatory factor 1 (EDRF1) are difficult and unsuccessful on many occasions. EDRF1 was identified as an interacting protein of dengue virus protease (NS3). The amino acid sequence analysis indicates the presence of NS3 cleavage sites in this protein. As EDRF1 is a high-molecular-weight (~138 kDa) protein, it is difficult to express and purify the complete protein. In this protocol, we clone the domain of the EDRF1 protein (C-terminal end) containing the cleavage site and the NS3 into two different eukaryotic expression vectors containing different tags. These recombinant vectors are co-transfected into mammalian cells. The cell lysate is subjected to SDS-PAGE followed by western blotting with anti-tag antibodies. Data suggest the disappearance of the EDRF1 band in the lane co-transfected along with NS3 protease but present in the lane transfected with only EDRF1, suggesting EDRF1 as a novel substrate of NS3 protease. This protocol is useful in identifying the substrates of viral-encoded proteases using ex vivo conditions. Further, this protocol can be used to screen anti-protease molecules.

Key features

• This protocol requires the cloning of protease and substrate into two different eukaryotic expression vectors with different tags.

• Involves the transfection and co-transfection of both the above recombinant vectors individually and together.

• Involves western blotting of the same PVDF membrane containing total proteins of the cell lysate with two different antibodies.

• Does not require purified proteins for the analysis of cleavage of any suspected substrate by the protease.

**Graphical overview**


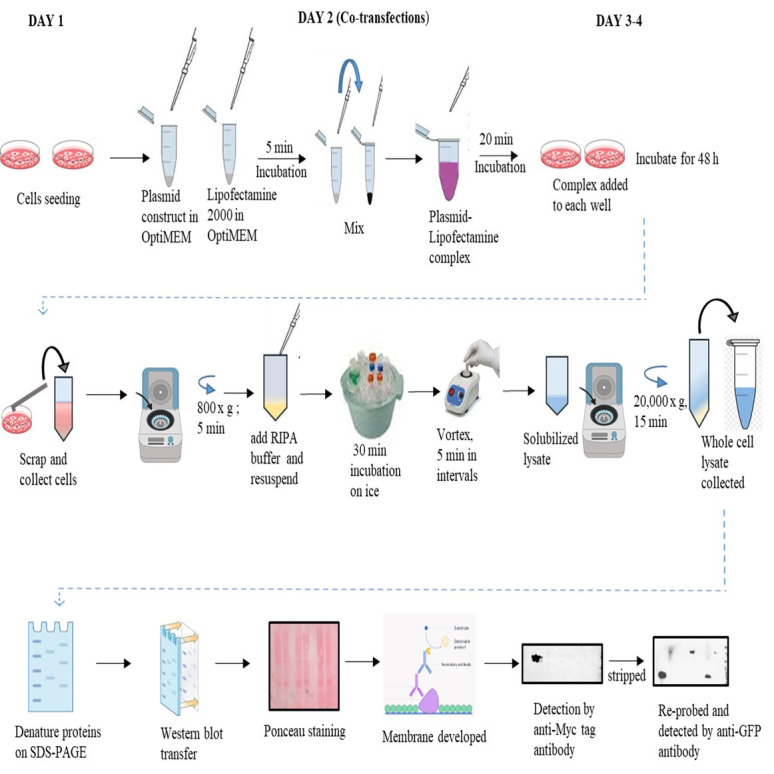


## Background

Viral encoded proteins mitigate cellular activities in order to bring the host under their control. In this direction, proteases of viral origin cleave the host proteins and cause irreparable damage to the host cells, hence being prime drug targets. Poliovirus-encoded 2A protease [1] cleaves the eukaryotic initiation factor 4G (eIF4G) and stalls cellular mRNA translation. Proteases of hepatitis C virus (HCV) [2] and dengue virus [3] are known to target the mitochondrial homeostasis, which leads to mitochondrial dysfunction and failure of the immune response. The main proteases 3CLpro and the papain-like protease (PLPro) of SARS CoV2 [4] possess protease activity, and the substrates need to be characterized. For this purpose, in silico techniques are useful, but experimental evidence is needed to confirm the analysis. In vitro pull-down assays or immunoprecipitation (IP) assays are being used to identify the novel substrates of viral-encoded proteases. 2D gel electrophoresis followed by MALDI-TOFF is also being used to this purpose but often yields false positives or negatives.

Dengue virus infections are hyperendemic in more than 130 countries across the globe, causing millions of infections and thousands of deaths annually. There is no vaccine or specific drugs developed to date, in spite of several attempts. One of the reasons for this is the failure to identify suitable drug/vaccine targets. The genome of this virus encodes three structural and seven non-structural (NS) proteins, along with two untranslated regions, one at each end. Among NS proteins that play significant roles during viral replication, NS3, alone or along with NS2B, possesses a crucial role and is the prime drug target for developing antivirals. NS3 is a 70 kDa protein that contains a 180 amino acid N-terminal trypsin-like serine protease domain followed by a C-terminal helicase domain [5]. This protein is a multifunctional serine protease, forming the catalytic triad with amino acids histidine (H-51), aspartate (D-75), and serine (S-135). NS3 is also known to regulate several host proteins to induce and maintain pathogenesis. Along with cleavage of the self-polypeptide to yield functional proteins, this protease is also known to cleave the host cellular proteins FAM134B (endoplasmic receptor), Ikα/β (cellular factors), nucleoporins (Nups), MITA, MFN1, and MFN2, thereby enhancing viral replication and affecting host metabolism [6-10]. However, there is still a lack of knowledge regarding the full list of host proteins that NS3 cleaves and its biological effects, particularly with regard to pathogenicity. In vitro cleavage experiments are useful to characterize such substrates but require purified proteins. In addition, the in vitro conditions sometimes fail to mimic the natural conditions and lead to erroneous results. NS2B–NS3 interactions have been targeted for drug development [11]. Therefore, this protocol is expected to be useful in the quick screening of anti-protease molecules against NS2B–NS3 interactions. The present protocol, an ex vivo study, describes the co-transfection followed by western blotting in order to evaluate EDRF1 as a novel substrate of dengue virus protease. Furthermore, this protocol is expected to be useful in the identification of any such substrates using NS3 of any of the four serotypes of dengue virus infections.

## Materials and reagents

All materials and reagents listed below can be acquired from other suppliers. In our lab, we use molecular and cell culture grade for all reagents. High-quality products are recommended for the cell culture experiments.

**Biological materials**

1. Cell lines: human embryonic kidney 293 cells (HEK 293), obtained from National Centre for Cell Science (NCCS), Pune, India

**Reagents**

1. Anti-GFP (D5.1) rabbit mAB (monoclonal antibody) (Cell Signalling Technology, catalog number: 2956)
2. Anti-Myc tag mouse monoclonal antibody (Proteintech, catalog number: 60003-2-Ig)
3. Rabbit anti-mouse-IgG HRP conjugated antibody (GeNei, catalog number: 1140580011730)
4. Mouse anti-rabbit-IgG HRP conjugated antibody (Santa Cruz Biotechnology, catalog number: sc-2357)
5. Dulbecco’s modified Eagle’s medium (DMEM) (Gibco, catalog number: 11995-065)
6. Fetal bovine serum (FBS) (Gibco, catalog number: 10270106)
7. Antibiotic (penicillin and streptomycin) (Gibco, catalog number: 15240062)
8. Trypsin-EDTA (1×) solution (HiMedia, catalog number: TCL007)
9. Lipofectamine 2000 (Invitrogen, catalog number: 11668-027)
10. Radio Immunoprecipitation Assay (RIPA) buffer (Sigma Aldrich, catalog number: R0278)
11. Femto LUCENT™ PLUS-HRP (G-Biosciences, catalog number: 786-003)
12. Protease inhibitor cocktail (Protease Arrest™) 100× (G-Biosciences, catalog number: 786-331)
13. Bradford reagent (Bio-Rad, catalog number: 5000201)
14. Immobilon^®^-P PVDF membrane (Merck Millipore, catalog number: IPVH00010)
15. Trypan blue (Sigma, catalog number: T6146)
16. OptiMEM (Gibco, catalog number: 31985062)
17. Ponceau S stain (Sigma, catalog number: P3504)
18. Tris base (HiMedia, catalog number: TC072)
19. Glycine (Merck, catalog number: 1.94907.0521)
20. Sodium dodecyl sulphate (SDS) (SR Lifesciences, catalog number: 14374)
21. NaCl (Merck, catalog number: 1.93206.0521)
22. KCl (HiMedia, catalog number: 7447-40-7)
23. NaH_2_PO_4 _(Merck, catalog number: MB024)
24. KH_2_PO_4 _(HiMedia, catalog number: PCT0009)
25. BSA (HiMedia, catalog number: GRM3151)
26. Skimmed milk powder (HiMedia, catalog number: GRM1254)
27. Tween-20 (SR Life Sciences, catalog number: 28599)
28. Absolute ethanol (Analytic CS, catalog number: 64-17-5)
29. Phosphate buffer saline (PBS) (Gibco, catalog number: 10010023)
30. GeneJet Plasmid MidiPrep kit (Thermo Scientific, catalog number: K0481)

**Solutions**

1. 10× PBS (see Recipes)
2. PBST (1× PBS and Tween-20) (see Recipes)
3. Blocking buffer (see Recipes)
4. 70% ethanol (see Recipes)
5. 10× transfer buffer (see Recipes)
6. Stripping buffer (see Recipes)

**Recipes**

1. **10× PBS**
  **Table:** 

Reagent	Final concentration	Quantity
NaCl	1.37 M	80 g
KCl	27 mM	2 g
NaH_2_PO_4_ KH_2_PO_4_	100 mM 18 mM	14.4 g 2.4 g
H_2_O	-	Up to 1 L
*Note: Adjust pH to 7.4. Stock can be stored at 25 °C up to one month.*
*Note: From this 10× stock, 500 mL of 1× PBS solution can be prepared: mix 50 mL of 10× PBS with 450 mL of H_2_O (can be stored at 25 °C for a week). Use double-distilled water (ddH_ 2_O) (autoclaved) for preparing 1× PBS solution.*
2. **PBST**
  **Table:** 

Reagent	Final concentration	Quantity
10× PBS Tween-20 H_2_O	1× 0.1% n/a	50 mL 500 μL 450 mL
*Note: Use ddH_2_O.*
3. **Blocking buffer**
  **Table:** 

Reagent	Final concentration	Quantity
BSA/skimmed milk powder PBST	2% or 7% 1×	0.2 or 0.7 g 10 mL
*Note: Prepare fresh when required. 7% blocking buffer solution is required for the blocking the PVDF membrane.*
*Optional: 2% blocking buffer solution is required for preparing antibody dilutions.*
4. **70% ethanol**
  **Table:** 

Reagent	Final concentration	Quantity
Ethanol H_2_O	70% n/a	350 mL 50 mL
*Note: Prepare fresh when required.*
5. **10**× **transfer buffer**
  **Table:** 

Reagent	Final concentration	Quantity
Tris	25 mM	30.2 g
Glycine	192 mM	144 g
H_2_O	-	up to 1 L
*Note: From this 10× stock, 1× transfer buffer can be prepared: dissolve 100 mL of 10× transfer buffer in 200 mL of methanol and make up the volume with water up to 1 L. Store at 4 °C; this can be reused for two weeks.*
6. **Stripping buffer**
  **Table:** 

Reagent	Final concentration	Quantity
Glycine	199 mM	1.5 g
SDS	0.1%	0.1 g
Tween-20	1%	1 mL
H_2_O		up to 100 mL
*Note: Adjust to pH 2.2.*

**Laboratory supplies**

1. Polypropylene conical-bottom centrifuge tubes, 15 and 50 mL Falcon (Tarsons, catalog number: 546021 and 546041) (sterile)
2. T-25 cell culture flasks (Tarsons, catalog number: 950040)
3. 60 mm cell culture dishes (Tarsons, catalog number: 960020)
4. Pipettes (1000, 200, and 20 μL) (Eppendorf, catalog numbers: 3123000063, 3123000055, 3123000098) and tips (Tarsons, catalog numbers: 523104, 523101, 523109)
5. Hand gloves (Kimberlay Gloves, catalog number: KC500-S)
6. 1.5 mL Eppendorf tubes (Tarsons, catalog number: 500010) and CRYOCHILL™ Vial 2D Coded (Tarsons, catalog number: 883192)
7. Parafilm 4" × 125' (SR Lifesciences, catalog number: 000814)
8. Aluminum foil
9. Discard box (any supplier)
10. Spray bottles (any supplier)
11. Cell scrapper (Tarsons, catalog number: 960051)

## Equipment

-80 °C deep freezer (Eppendorf, catalog number: F570)
2. Microcentrifuge (Eppendorf, catalog number: 5424R)
3. CO_2_ incubator (Eppendorf, catalog number: Galaxy 48R)
4. Inverted brightfield microscope (Lawrence and Mayo, catalog number: TC5400)
5. Chemidoc image analyzer (Bio-Rad, catalog number: 12003028)
6. Electroblot transfer unit (BioNova)
7. SDS-PAGE unit (Bio-Rad, model: Mini-PROTEAN Tetra Cell, catalog number: 1658038)
8. Hemocytometer (Neubeur Blood Counting Chamber)
9. Laminar hood (Laminar Flow Systems)
10. Animal cell culture facility
11. Autoclave (KETAN, catalog number: PA21)
12. Water bath (GeNei, catalog number: 107931GB)
13. Vortex mixer (Tarsons, catalog number: 3002)

## Software and datasets

1. Image Lab software (Bio-Rad) (v6.1.0, 2020)
2. Zen software for Carl Zeiss fluorescence microscope (Zen 2.3, blue edition, v2.3.69.1000)

## Procedure

- **Culturing and maintenance of cells**
  1. Take a 2.0 mL cryovial of HEK 293 cells from -80 °C or liquid nitrogen.
    **Caution:** Use cryogenic hand gloves for taking the vial from liquid nitrogen or -80 °C.
    Thaw the cryovial in a water bath for 2–3 min at 37 °C.
  2. Add 1 mL of serum-free DMEM to the thawed vial.
    - Collect the cells and transfer in a 15 mL Eppendorf tube.
    - Centrifuge the cells at 800*× g* for 3 min.
    - Replace the medium with 1 mL of fresh complete DMEM [containing heat-inactivated 10% FBS and 1% antibiotic (v/v) (penicillin and streptomycin)].
  3. Count ~8 *×* 10^5^ cells using a hemocytometer and seed in T-25 flask.
  4. Incubate the cells at 37 °C in a humidified incubator for 12–16 h with 5% CO_2_.
    - The next day, observe the cells under the inverted brightfield microscope for their proper adherence (Figure 1 ).
    - Replace the medium with 3 mL of fresh complete DMEM (containing 10% FBS and 1% antibiotics).
    **Figure 1.**
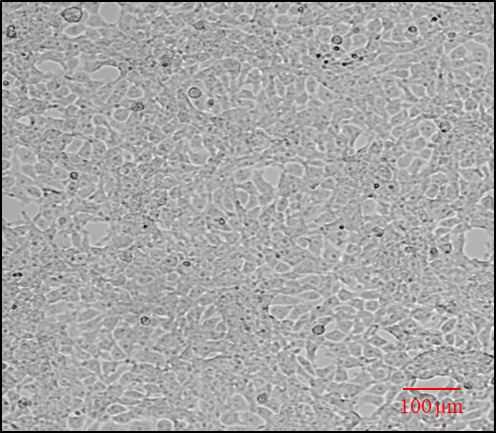
Representative image showing HEK cell confluency (70%–80%) and adherence analyzed under brightfield inverted microscope
- **Cell splitting, counting, and seeding**
  1. From the above cultured T-25 flask, discard the medium.
    - Add 1 mL of Trypsin-EDTA solution (1×) and incubate for 3 min at 37 °C in a CO_2_ incubator.
    - Observe the detached cells under the brightfield inverted microscope.
    - Once the cells are detached, add 1 mL of complete DMEM to inactivate the trypsin and collect cells into a fresh 15 mL Falcon tube.
      **Optional:** Add 1 mL of serum-free DMEM to collect the leftover cells from T-25 flasks.
    - Harvest cells by centrifugation at 500*× g* for 3 min.
    - Wash the cell pellet with 1 mL of serum-free DMEM medium (or mix 500 μL of DMEM and 500 μL of 1× PBS in 1:1 ratio). Resuspend the cells gently by pipetting up and down.
    - Centrifuge again at 500*× g* for 3 min.
    - Discard the medium, add 1 mL of fresh complete DMEM medium, and suspend gently.
    - Prepare two or three T-25 flasks with 3 mL of complete DMEM medium and add 200 μL of the above resuspended cell suspension (in ratios 1:10 or 1:15) to the flasks.
    - Gently shake the flasks (back and forth) and allow cells to grow in a humidified CO_2_ incubator at 37 °C.
  2. To perform counting of the cells, follow the above steps B1a–g.
  3. Take 1 mL of cell suspension and count using a hemocytometer with trypan blue.
    - In a fresh 1.5 mL Eppendorf tube, add 100 μL of trypan blue dye and 100 μL of cell suspension. Mix gently with a pipette.
    - Place the coverslip on the hemocytometer and add 10 μL of trypan blue cell suspension under the coverslip using a pipette. Allow the cells to spread evenly on the counting chamber.
    - Count the number of viable cells from the four big squares of the hemocytometer using the formula:
      No. of cells/mL = (No. of cells in 4 big squares/4) × 10^4 ^× dilution factor*
      *Dilution factor = 2 (100 μL of trypan blue dye: 100 μL of cell suspension)
      10^4^ = Conversion factor to 1 mL
      For example:
      No. of cells in 4 big squares: 1000
      No of cells/mL = (1000/4) x 10^4 ^× 2 i.e., 5 × 10^6 ^cells/mL
      To seed 1 × 10^6^ cells/mL, add 200 μL of cell suspension in 800 μL of complete DMEM.
  4. Seed 1 × 10^6^ cells per dish (60 mm dishes) and allow to grow to attain ~80% confluency at 37 °C in a CO_2_ incubator for 12–14 h (see Troubleshooting 4).
- **Co-transfection**
  1. Observe the cells seeded in 60 mm dishes in the above step to confirm confluency (70%–80%).
  2. Use the fresh plasmids (pcDNA3.1 c-myc, pEGFP-N1 vectors, and the recombinant vectors, i.e., pcDNA3.1 c-myc EDRF1 and pEGFP-N1 NS2BNS3pro) for transfection (Please see supplementary Figures S1A and B for the recombinant construct development).
*Note: Isolate the plasmids using MidiPrep two days before the transfection experiment. Prepare 20–30 μL of aliquots in 0.5 mL tubes and store at -20 °C.*
  3. Take 1.5 mL Eppendorf tubes, add 1 μg of pcDNA3.1 c-myc vector + 1 μg of pEGFP-N1 vector (tube 1), 1 μg of pcDNA3.1 c-myc EDRF1 (tube 2), 1 μg of pcDNA3.1 c-myc vector (tube 3), 1 μg of pcDNA3.1 c-myc EDRF1 + 1 μg of pEGFP-N1 NS2BNS3pro (tube 4), 1 μg of pEGFP-N1 vector (tube 5), and 1 μg of pEGFP-N1-NS2BNS3pro (tube 6) in OptiMEM (Mix-1) (see Troubleshooting 5).
    **Optional:** Any other vector containing two different tags can also be used for transfection (FLAG or HA tag vectors).
    - Incubate the OptiMEM-diluted plasmids for 2–3 min at room temperature.
  4. Take separate 1.5 mL tubes and add 2 μL and 4 μL of the Lipofectamine 2000 in OptiMEM (Mix-2).
    - Incubate the OptiMEM-diluted Lipofectamine for 2–3 min at room temperature.
    See Table 1 for an example.
    **Table 1.** Preparation of transfection and co-transfection reaction mixtures using Lipofectamine 2000

		Mix-1		Mix-2	
S. No	Transfection/ Co-transfection	Quantity of plasmids	Volume of OptiMEM (μL)	Volume of Lipofectamine (μL)	Volume of OptiMEM (μL)
1.	pcDNA3.1 c-myc vector + pEGFP-N1 vector	2 μL (1 μg) + 2 μL (1 μg)	46	4	46
2.	pcDNA3.1 c-myc EDRF1	2 μL (1 μg)	48	2	48
3.	pcDNA3.1 c-myc vector	2 μL (1 μg)	48	2	48
4.	pcDNA3.1 c-myc EDRF1 + pEGFP-N1 NS2BNS3pro	2 μL (1 μg) + 2 μL (1 μg)	46	4	46
5.	pEGFP-N1 vector	2 μL (1 μg)	48	2	48
6.	pEGFP-N1-NS2BNS3pro	2 μL (1 μg)	48	2	48
*Note: Each plasmid (1 μg) with lipofectamine (2 μL) is in 1:2 ratio. Plasmid to Lipofectamine can also be used in ratios 1:3, 1:4, and 1:5. In the co-transfected condition, the total plasmid concentration is 2 μg (1 μg of each plasmid), so 4 μL of Lipofectamine is used.*
  5. Add Mix-1 to Mix-2 and incubate for 20 min to form a plasmid–Lipofectamine complex.
  6. Add the complex by gentle pipetting onto the cells drop by drop and further incubate the cells at 37 °C in a CO_2_ incubator for 5–6 h.
  7. Replace the media with fresh complete DMEM and allow the cells to grow for 48 h.
  8. After 48 h of transfection, remove the media from the plates and add 1 mL of 1× PBS.
    - Scrap the cells with a cell scrapper and collect the cells by pipetting into a fresh 1.5 mL Eppendorf tube.
    - Centrifuge at 800× *g* for 5 min.
    - Wash the cells with 1 mL of 1× PBS and centrifuge at 800*× g* for 5 min.
    - Repeat the wash step twice if pelleted cells still contain traces of DMEM medium.
  9. Add 200 μL of RIPA buffer and 20 μL of 1× Protease inhibitor cocktail to the cell pellet and resuspend gently.
    - Vortex the suspension thrice at 5 min intervals and keep on ice for 30 min.
    - Centrifuge the cell suspension at 20,000× *g* for 15 min.
    - Collect the supernatant into a fresh Eppendorf tube as whole-cell lysate.
  10. Quantify the total protein of the lysates by Bradford reagent.
    - Resolve 60 μg of the quantified protein on 10% SDS PAGE.
  **Optional:** Before proceeding with the cell harvesting step C8, GFP expression can be analyzed using a fluorescence microscope.
*Note: GFP expression was analyzed to confirm the transfection.*
- **Western blotting**
  1. Transfer the above resolved proteins onto PVDF membrane at 80 V current for 3 h (or 50 V overnight) using a western blot transfer unit in 1× transfer buffer.
  2. Stain the PVDF membrane with Ponceau S stain.
    - Prepare 50 mL of fresh Ponceau S stain solution.
    - Add 4–5 mL of Ponceau S stain onto the PVDF membrane and immerse the membrane completely.
    - Incubate the membrane for 5 min with slow agitation on the rocker.
    - Remove the Ponceau S stain and collect it in an Eppendorf tube for reuse (at least twice).
    - Add ddH_2_O water to wash out the excess stain and, as the bands start appearing immediately, record the image.
  3. Wash the membrane with 1× PBST until the Ponceau is removed completely.
*Note: Ponceau stain must be removed as it may hinder the blocking step.*
  4. Prepare 7% blocking buffer and block the membrane for 2 h at room temperature under gentle shaking.
*Note: Blocking step needs to be optimized for each antibody. 7% Blocking buffer is used to avoid non-specificity.*
  5. Rinse the membrane once with 1× PBST and add the primary antibody solution [anti-Myc Tag (1.4 μg/mL, 1:1000) in 1× PBST or 2% blocking buffer solution].
*Note: 2% Blocking buffer is used for diluting the primary antibodies.*
    - Incubate the membrane at room temperature for 2 h or at 4 °C overnight with gentle rocking.
*Note: Incubation time and dilution of antibody need to be optimized based on the non-specific bands.*
  6. Wash the membrane with 1× PBST three times for 10 min each on a rocker with mild agitation.
  7. Add secondary antibody (anti-mouse IgG HRP conjugated; 1:10,000) diluted in 1× PBST.
  8. Incubate the membrane for 2 h at room temperature.
  9. Wash the membrane with 1× PBST three times for 15 min each with high agitation.
  10. Develop the washed membrane with western blot Femto LUCENT™ PLUS-HRP substrate solution and capture the image using Chemidoc Image System (Bio-Rad) (see Troubleshooting 6).
- **Stripping and re-probing the PVDF membrane**
  Stripping is performed to re-probe the membrane with the different antibodies. All the steps for stripping can be carried out at room temperature.
  1. From the above step D9, use the membrane for stripping.
    - Wash the membrane with ddH_2_O two times for 5 min each on the rocker with medium agitation.
    - Repeat the wash step two times with 1× PBST for 5 min each on the rocker with medium agitation.
    - Add ~10 mL of stripping buffer and incubate the membrane for 30 min on the rocker with medium agitation at room temperature.
    - Discard the stripping buffer and wash the membrane with ddH_ 2_O for 5 min on a rocker.
    - Wash the membrane with 1× PBS two times for 5 min each on the rocker.
    - Repeat the wash steps with 1× PBST two times for 10 min each on the rocker.
    - The membrane is ready for the re-probing with different antibodies.
  2. For re-probing with the different antibodies, begin with the blocking steps as mentioned in the above western blotting steps (D4–D9).
*Note: For re-probing, in our experiment, we have used anti-GFP antibody (1:1,000) (primary antibody) and anti-rabbit HRP conjugated antibody (0.04 μg/mL, 1:10,000) (secondary antibody).*

## Data analysis

**Figure 2.**
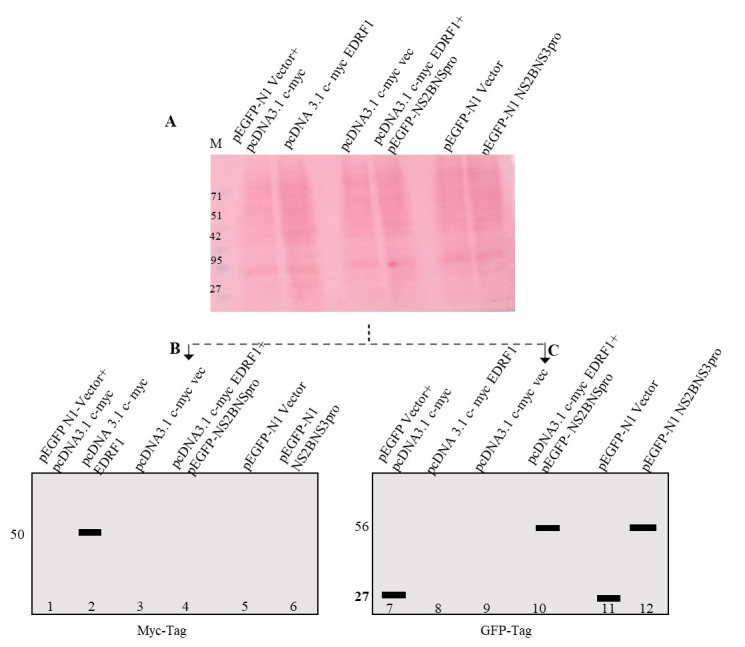
Western blotting analysis of co-transfected lysates. A. Ponceau image after electroblot transfer. The lysate loaded is indicated on each lane. B. and C. Simulated images of western blotting probed with anti-Myc and anti-GFP antibodies.

In this protocol, we analyzed EDRF1 cleavage by co-transfection followed by western blotting. The western blot data was obtained using anti-Myc tag antibody for pCDNA 3.1 c-myc EDRF1 and GFP-tag antibody for pEGFPN1 NS2BNS3pro expressions. In pcDNA3.1 c-myc vector containing EDRF1 as an insert, the EDRF1 band was detected as intact showing the presence of expressed EDRF1 alone (Figure 2, lane 2). As expected, no expression was observed in pcDNA3.1 c-myc vector alone. Importantly, the expressed EDRF1 completely disappeared in the presence of protease in co-transfected pCDNA3.1 c-myc EDRF1 and pEGFP-N1 NS2BNS3pro conditions, suggesting EDRF1 as a substrate of protease (Figure 2, lane 4). pEGFP-N1 vector alone and pEGFP-N1 NS2BNS3pro lysates were loaded as controls (Figure 2, lanes 11 and 12). To confirm the expression of NS2BNS3pro in the above experiment, we have checked the expression of protease using anti-GFP antibody (pEGFPN1-NS2BNS3pro) after stripping the same membrane. It was observed that, in co-transfected vectors and pEGFP-N1 vector alone, GFP was expressed. In pcDNA3.1 c-myc EDRF1 and pEGFP-N1 NS2BNS3pro co-transfected cell lysates, the protease was detected, thus confirming the expression of NS2BNS3pro in the co-transfected conditions (Figure 2, lane 10). pEGFP-N1 NS2BNS3pro was also found to be expressed alone, which is a control (Table 2). We expect that this analysis will be useful in identifying the novel substrates of viral encoded proteases, as this protocol is simple, easy, and quick to perform. Further, this protocol can be used to evaluate anti-protease molecules.

**Table 2.** Tabulated summary of the data

		Myc-Tag antibody	GFP-Tag antibody
S. No	Plasmid Transfected	Myc-tag fusion protein	GFP-tag fusion protein
1.	pcDNA 3.1 c-myc vector + pEGFP-N1 vector	No bands will be detected as the Myc tag is 1.2 kDa (co-transfected empty vectors)	GFP protein will be detected due to the presence of pEGFP-N1 vector ~27 kDa (co-transfected empty vectors)
2.	pCDNA 3.1 c-myc EDRF1	~ 50 kDa pcDNA3.1c-myc EDRF1 detected	No band detected
3.	pcDNA 3.1 c-myc vector	No band detected	No band detected
4.	pcDNA 3.1 c-myc EDRF1 + pEGFP-N1 NS2BNS3pro	No band detected due to cleavage of EDRF1 in presence of protease (co-transfected)	~56 kDa pEGFP-N1 NS2BNS3pro band detected showing the presence of GFP tag protease (co-transfected)
5.	pEGFP-N1vector	No band detected	~ 27 kDa GFP band detected
6.	pEGFP-N1 NS2BNS3pro	No band detected	~56 kDa pEGFP-N1 NS2BNS3pro band detected

## Validation of protocol

This protocol was described in our published article in iScience (2023), https://doi.org/10.1016/j.isci.2023.107024. We have repeated the protocol and found that it is easy to execute, and the outcome is consistent. *In vitro* pulldown assay followed by mass spectrometry identification/western blotting confirmed the interaction between EDRF1 and protease. *In vitro* sequence analysis suggested the existence of a total of five protease cleavage sites in EDRF1. Further, superimposed models of EDRF1 and protease indicated the location of the cleavage site within the catalytic triad of protease. The above observations support the outcome of the protocol described.

## General notes and troubleshooting

**General notes**

This protocol requires either the two differently expressing vectors with different tags or proteins specific for analyzing the expression, if using the same tag for the co-transfected plasmids. The protocol is more convenient to be performed with two different tagged vectors.

**Troubleshooting**

**Table:** 

Potential problem	Possible cause	Corrective measures
1. Cell line contamination and cross contamination	Presence of bacterial/fungal/mycoplasma. Cell line obtained from other labs. Improper handling during cell culture maintenance of two cell lines together. Preparation of cryovial with contaminated cryomedium.	Proper use of sterile equipment and reagents in cell culture will allow maintenance of the aseptic environment and minimize contamination. Collect the cells from the certified cell repositories (NCCS, Pune, India, or ATTC). Culture and split the cells one at a time or perform the splitting of cells on alternate days. Prepare the cryomedium fresh, if possible. Use cell culture grade DMSO or glycerol or commercially available cryomedium. Check the expiry date of the FBS, media, and antibiotics used for culturing the cells.
2. Cell culture media color changes	Highly confluent dishes. CO_2_ levels are low. Bacterial contamination.	Thaw a new cryovial or split the cells as needed. Set the CO_2_ levels to 5% and maintain humidity. Add antibiotics and wash the cells with 1× PBS (cell culture grade). Discard the media and sterilize the laminar hood cabinets and CO_2 _incubator.
3. Cells did not attach after being obtained from cryogenic stage	Cryomedium shows toxicity. Too many apoptotic cells. Less cell numbers during cryofreezing. Higher number of continuous passages.	Use 5%–7% DMSO in cryomedium. Use 90% FBS during cryofreezing. Prepare cryovials with 80% confluent freshly split cells.
4. Cell growth slows	Less or inaccurate supplemental cell culture components. Cell culture conditions (temperature, humidity, and CO_2_ levels). Protein of interest not expressing.	Begin with fresh cryovials. Growth media with 10%–12% FBS will be good for attaining a good confluency. Keep the incubator at 37 °C with 5% CO_2 _and water tray. Grow the cells without antibiotics.
5. Co-transfection not working	Transfection reagents may not be suitable. Cell lines may not be efficient for co-transfection. Co-transfecting plasmids not expressing together.	Check the quantity and quality of plasmids. Use high quality plasmid isolation kits (Thermo MidiPrep or High Pure Links Kits) Lipofectamine 2000 works best for most cell lines. Lipofectamine 3000 can also be used. Check the transfection efficiency before proceeding with the co-transfection experiments. Perform and analyze the transfection of the individual plasmids that need to be co-transfected.
6. Western blot of the co-transfected plasmids	Antibody not detecting the proteins in co-transfecting plasmids. No signal in the blot. High background.	Optimize the transfection timing for the co-transfected plasmids. Forty-eight hours of co-transfection give conclusive results. Use individual plasmids as controls to confirm the transfection. Optimize the antibody dilution (1:500 to 1:3,000). Use specific tagged antibodies or protein-specific antibodies. Use species-specific secondary antibodies (anti-mouse or anti-rabbit HRP conjugated). Optimize the primary and secondary antibody incubation times and washing steps. Optimize the blocking buffer (5%–7% BSA or skimmed milk). Use 1× PBST and up to 0.3% Tween-20.

## Supplementary information

The following supporting information can be downloaded here:

1. Figure S1A: Cloning and expression of DENV protease ex vivo.
2. Figure S1B: Cloning and expression of EDRF1 ex vivo.
